# Scholarly Productivity Evaluation of KL2 Scholars Using Bibliometrics and Federal Follow-on Funding: Cross-Institution Study

**DOI:** 10.2196/29239

**Published:** 2021-09-29

**Authors:** Kelli Qua, Fei Yu, Tanha Patel, Gaurav Dave, Katherine Cornelius, Clara M Pelfrey

**Affiliations:** 1 Clinical and Translational Science Collaborative School of Medicine Case Western Reserve University Cleveland, OH United States; 2 Health Sciences Library University of North Carolina at Chapel Hill Chapel Hill, NC United States; 3 School of Information and Library Science University of North Carolina at Chapel Hill Chapel Hill, NC United States; 4 North Carolina Translational and Clinical Sciences Institute School of Medicine University of North Carolina at Chapel Hill Chapel Hill, NC United States; 5 Division of General Medicine and Clinical Epidemiology School of Medicine University of North Carolina at Chapel Hill Chapel Hill, NC United States; 6 Center for Clinical and Translational Science Mayo Clinic Rochester, MN United States

**Keywords:** bibliometrics, Clinical and Translational Science Award, KL2, translational research, career development

## Abstract

**Background:**

Evaluating outcomes of the clinical and translational research (CTR) training of a Clinical and Translational Science Award (CTSA) hub (eg, the KL2 program) requires the selection of reliable, accessible, and standardized measures. As measures of scholarly success usually focus on publication output and extramural funding, CTSA hubs have started to use bibliometrics to evaluate the impact of their supported scholarly activities. However, the evaluation of KL2 programs across CTSAs is limited, and the use of bibliometrics and follow-on funding is minimal.

**Objective:**

This study seeks to evaluate scholarly productivity, impact, and collaboration using bibliometrics and federal follow-on funding of KL2 scholars from 3 CTSA hubs and to define and assess CTR training success indicators.

**Methods:**

The sample included KL2 scholars from 3 CTSA institutions (A-C). Bibliometric data for each scholar in the sample were collected from both SciVal and iCite, including scholarly productivity, citation impact, and research collaboration. Three federal follow-on funding measures (at the 5-year, 8-year, and overall time points) were collected internally and confirmed by examining a federal funding database. Both descriptive and inferential statistical analyses were computed using SPSS to assess the bibliometric and federal follow-on funding results.

**Results:**

A total of 143 KL2 scholars were included in the sample with relatively equal groups across the 3 CTSA institutions. The included KL2 scholars produced more publications and citation counts per year on average at the 8-year time point (3.75 publications and 26.44 citation counts) than the 5-year time point (3.4 publications vs 26.16 citation counts). Overall, the KL2 publications from all 3 institutions were cited twice as much as others in their fields based on the relative citation ratio. KL2 scholars published work with researchers from other US institutions over 2 times (5-year time point) or 3.5 times (8-year time point) more than others in their research fields. Within 5 years and 8 years postmatriculation, 44.1% (63/143) and 51.7% (74/143) of KL2 scholars achieved federal funding, respectively. The KL2-scholars of Institution C had a significantly higher citation rate per publication than the other institutions (*P*<.001). Institution A had a significantly lower rate of nationally field-weighted collaboration than did the other institutions (*P*<.001). Institution B scholars were more likely to have received federal funding than scholars at Institution A or C (*P*<.001).

**Conclusions:**

Multi-institutional data showed a high level of scholarly productivity, impact, collaboration, and federal follow-on funding achieved by KL2 scholars. This study provides insights on the use of bibliometric and federal follow-on funding data to evaluate CTR training success across institutions. CTSA KL2 programs and other CTR career training programs can benefit from these findings in terms of understanding metrics of career success and using that knowledge to develop highly targeted strategies to support early-stage career development of CTR investigators.

## Introduction

Evaluating outcomes of a Clinical and Translational Science Award (CTSA) hub’s clinical and translational research (CTR) training, such as the KL2 program, requires the selection of reliable, accessible, and standardized measures. The KL2 program is a multiyear mentored training award focusing on early-stage career development of investigators in CTR. The National Center for Advancing Translational Science (NCATS) at the National Institutes of Health (NIH) funds over 60 KL2 programs across CTSA hubs. All CTSA hubs offer a KL2 program, which is a formal, mentored training experience for scholars with doctoral degrees. Each CTSA hub selects KL2 candidates from a variety of fields (eg, medicine, nursing, and biostatistics) to participate in translational research training in a multidisciplinary setting with up to 5 years of career development support.

One of the strategic goals of NCATS for the CTSA program is to “develop and foster innovative translational training and a highly skilled, creative, and diverse translational science workforce” [1]. The KL2 program is one mechanism that offers such training. Each CTSA hub is responsible for evaluating its KL2 program, typically using methods such as surveys, focus groups, exit interviews, and alumni follow-up. Measures of scholarly success are predominantly scholarship products—publication output and extramural funding, such as an R01-equivalent award. The NCATS Common Metrics Initiative also created measures for all KL2 programs to report on the number and percentage of total graduates: women, underrepresented minorities, and KL2 scholars who sustain their translational research engagement [2].

All NCATS-funded KL2 programs follow a set of established requirements. For example, 75% of the time of enrolled KL2 scholars is funded by the program, with the exception of 50% for surgeons [3]. All KL2 programs must provide training in rigorous research methodologies aligned with CTR competencies. KL2 programs must provide opportunities that allow scholars to effectively communicate and collaborate across multidisciplinary teams [3,4]. Beyond these requirements, CTSA hubs are encouraged to innovate and tailor their KL2 programs to their specific needs, leading to some variability in KL2 programs. For example, although programs provide 2 years of KL2 funding, some programs provide funding for 3 or more years [5]. Such differences in hub-level KL2 program characteristics present challenges for defining indicators of success and standardizing the evaluation of KL2 program outcomes across CTSA hubs.

CTSA hubs sometimes use bibliometric analysis to evaluate their impact on moving translational research forward. Bibliometrics refers to the use of quantitative and statistical methods to analyze a chosen group of publications [6,7]. Bibliometrics complement other evaluation methods, such as surveys and interviews, by allowing programs to measure tangible and intangible outcomes such as publications and scholarly impact, which otherwise cannot be objectively measured via self-report methods such as surveys or interviews. One study [8] used bibliometrics to analyze publications citing all CTSA hub grants from 2006 to 2016. This study highlighted bibliometric values to assess the impact of research across all CTSAs as measured by interdisciplinary collaboration, influence on other publications, and breadth of scientific fields. Another study [9] used bibliometrics to compare publications citing one of 6 CTSA hubs with measures from three sources: NIH iCite (publicly available), Thomson Reuters (now Clarivate Analytics), and Elsevier. This study identified relevant data sources and standardized analyses for cross-CTSA comparisons. A more recent CTSA hub-specific study [10] implemented advanced bibliometric measures to assess scholarly productivity, citation impact, the scope of research collaboration, and clusters of research topics of publications supported by their CTSA. Furthermore, some CTSA hubs have evaluated the feasibility of different bibliometric approaches to assessing KL2 scholarly productivity and influence compared with other NIH-funded K-awardees [11,12]. These studies illustrated the importance of standardizing the (1) collection of publication data, (2) definition of success and measures for KL2 training outcomes, and (3) use of bibliometric methods to evaluate the scholarly productivity and impact of KL2 scholars across the CTSA consortium.

To our knowledge, the evaluation of KL2 programs across CTSAs is limited, and the use of bibliometrics and follow-on funding is minimal. This study evaluates scholarly productivity using bibliometrics and federal follow-on funding of KL2 scholars across 3 different CTSA hubs to define and assess CTR training success indicators. Such indicators, coupled with evidence of the feasibility of their collection and use, would allow CTR training programs to demonstrate effectiveness on a wide variety of outcomes.

## Methods

### Participants

Our sample included KL2 scholars from 3 CTSA institutions, described as institutions A-C throughout the paper. A description of program-level characteristics is provided in the Multimedia Appendix 1. The 3 institutions were selected as convenience samples based on previous collaborations between the CTSAs. We only included scholars in our analysis if they had at least 5 years of bibliometric data, starting at matriculation (ie, year of entry into the KL2 program). We chose the date range of 2005-2013 to ensure that all scholars met the minimum 5-year data requirement. In other words, scholars beginning their KL2 program in 2013 have 5 years of bibliometric data as of December 2018.

### Measures

#### Overview

We selected bibliometrics and federal follow-on funding because they are objective measures with evidence of reliability and validity and are verifiable. In the following sections we describe various bibliometrics along a continuum of objectivity. Although some metrics, such as number of publications, are objective, other metrics, such as citation impact and collaboration, are quantified representations of construct and are open to interpretation of scale, scope, and reliability.

#### Bibliometrics

When we selected bibliometric measures and sources for this study, we considered the (1) validity, relevancy, and feasibility of measures, especially the metrics and sources that have been verified and adopted in previous CTSA evaluation studies; (2) coverage of publications of our KL2 scholars in three CTSAs; and (3) availability of bibliometric data sources such as institutional subscriptions and free accessibility. Accordingly, we chose Elsevier SciVal and NIH iCite for the bibliometric measurements. SciVal is a research analytics tool for various bibliometrics gathered from the Elsevier Scopus citation database. Scopus has much broader coverage in biomedical and life sciences than Web of Science [13,14] and has been adopted in multiple bibliometric studies concerning CTSA evaluations [9,10,15]. The 3 CTSA institutions in this study have subscriptions to Scopus or SciVal and have experience in using Elsevier metrics to assist research performance evaluation. In addition, we added the relative citation ratio (RCR) generated by NIH iCite as an additional citation measure. Being freely accessible and increasingly used by CTSA evaluators, RCR is a field-independent metric, measuring the citation impact of an article relative to other NIH-supported research papers produced in the same field during the same timeframe. However, iCite is limited to analyzing only articles indexed in PubMed, whereas SciVal can provide aggregated data from the more comprehensive Scopus database. Extracting data from both sources can help reduce potential bias and provide a more reliable estimate of the quantity and impact of scholarly work. Therefore, we exported bibliometric data for each of the included scholars from both SciVal and iCite to provide evidence of validity and reliability within this sample [16,17].

Data exported from SciVal provide evidence for three metrics of research performance: productivity, impact, and collaboration. Productivity metrics provide an overview of total scholarly output, that is, the number of publications a scholar produces within a specified period. Impact metrics focus on citation counts through raw and calculated variables, accounting for scientific field-weighted or ratio values. We excluded self-citations from the analysis. Collaboration metrics consider all authors in a publication with attention to the affiliated institutions of coauthors. We used iCite data to understand the impact of the publications compared with the average of NIH-funded publications published in the same year and field [18]. Table 1 shows a detailed list of metrics used per domain.

**Table 1:** Summary of bibliometrics used.

Source, domain, and metric			Definition
**SciVal**			
	**Productivity **		
		Scholarly output	Number of publications indexed in Scopus
	**Impact**		
		Citations per publication	Average number of citations received per publication
		PPTPJ^a^	Number of publications in the world’s top journals
		FWCI^b^	Ratio of citations received relative to the expected average for the field, type, and year
	**Collaboration**		
		NFWC^c^	Collaboration ratio is computed based on the expected collaboration for that document type, publication year grouping, and subject area assignment
**iCite**			
	**Impact**		
		Average RCR^d^	Cites per year of each paper, normalized to the citations per year received by NIH^e^-funded papers in the same field and year
		Average citations per year	Citations per year received since publication; this is the numerator for the RCR.
		Average field citation rate	Intrinsic citation rate of this paper’s field is estimated using its co-citation network.
		Average NIH percentile	Percentile rank of this paper’s RCR compared with all NIH publications.

^a^PPTPJ: percentage of publications in the top 10th percentile of journals.

^b^FWCI: field-weighted citation impact.

^c^NFWC: National Field-weighted Collaboration.

^d^RCR: relative citation ratio.

^e^NIH: National Institutes of Health.

#### Federal Follow-on Funding

Three federal follow-on grant funding measures were also collected: NIH funding at the 5-year, 8-year, and overall time points. These follow-on funding measures include only NIH federal funding received by a scholar as the principal investigator, coprincipal investigator, or coinvestigator. This information can be independently confirmed for all 3 institutions by examining NIH RePORTER (Research Portfolio Online Reporting Tools Expenditures and Results) for NIH funding records [19].

### Procedures

This study received institutional review board exemption from 2 institutions, with a third institution determining that this was not human subjects research. Each participating institution developed a list of KL2-scholars awarded since 2005, including gender, race, ethnicity, and highest degree earned for each scholar. We dichotomized race due to sample skewness; however, dichotomization still resulted in unequal group sizes. We excluded ethnicity analysis because of the homogeneity of the sample. We collapsed the categories of degrees to MD and non-MD terminal degrees. Clinical training was the distinction between MD and non-MD degrees. Scholars with both MD and PhD were considered MDs.

Cohorts of scholars were created in SciVal using the year the scholar began their KL2-grant (ie, all 2005 KL2’s were in one cohort) according to the SciVal cohort-instructions. To obtain iCite data, a list of publications associated with each KL2 scholar was retrieved from PubMed and then manually verified. The PubMed identification numbers (PMIDs) of the verified publications were filtered to the 2005-2013 date ranges for this analysis. Next, the validated PMIDs were imported into iCite to download the data for each scholar. All metrics from SciVal and iCite were extracted and entered into an SPSS (IBM; version 25) file within 6 months. Grant data were collected using internal records of federal funding and confirmed through NIH RePORTER [19].

### Data Analysis

All statistical analyses were computed using SPSS [20]. Nonparametric tests for independent samples (Kruskal-Wallis and Mann-Whitney U) were used when data were not normally distributed. We conducted descriptive and inferential statistical analyses according to demographic subgroups and institutions to assess the bibliometric results of KL2 scholars. Federal funding data were analyzed using logistic regression to evaluate the relationship between categorical variables (eg, gender, race, degree, and institution) and a series of dichotomized variables representing the presence or absence of federal funding at three time points, as described below.

Data were time-bound to include the year the scholar started their K-award (ie, matriculation) through the completion of 2018. Three time points determined analysis: 5 years and 8 years after matriculation and overall (from the start year of the KL2-award through the end of 2018). These time points are based on established precedents from K08 or K23 evaluations and K to R-award funding trajectories, which peak at 8 years [21,22]. iCite data were not included for the 5-and 8-year time points because, at the time of analysis, no time point–specific data could be produced by iCite and only the overall citation metrics were available.

## Results

### Sample Characteristics

The total sample consisted of 143 KL2 scholars with relatively equal groups across institutions (Table 2). The overall sample at each institution had a slightly greater number of male scholars. The majority of KL2 scholars in this sample were non-Hispanic White people. Most scholars held terminal MD degrees, with PhDs being the second most common degree.

**Table 2:** Summary of scholar demographics^a^.

Demographics			Sample value (N=143)		Institution A value (n=50)		Institution B value (n=48)		Institution C value (n=45)
**Gender, n (%)**									
	Male	*77 (54)^b^*		*27 (54)*		*25 (52)*		*25 (55)*	
	Female	66 (46)		23 (46)		23 (48)		20 (44)	
**Ethnicity, n (%)**									
	Hispanic or Latino	6 (4)		4 (8)		1 (2)		1 (2)	
	Not Hispanic or Latino	*136 (95)*		*45 (90)*		*47 (98)*		*44 (98)*	
	Not reported	1 (1)		1 (2)		0 (0)		0 (0)	
**Race, n (%)**									
	American Indian	0 (0)		0 (0)		0 (0)		0 (0)	
	Asian	19 (13)		9 (18)		1 (2)		9 (20)	
	African American	10 (7)		3 (6)		2 (4)		5 (11)	
	White	*112 (78)*		*36 (72)*		*45 (94)*		*25 (55)*	
	Not reported	1 (1)		2 (4)		0 (0)		0 (0)	
**Degree, n (%)**									
	MD	*74 (52)*		*20 (40)*		*29 (60)*		*25 (55)*	
	PhD	47 (33)		17 (34)		15 (31)		15 (33.3)	
	MD and PhD	15 (11)		7 (14)		4 (8)		4 (8.9)	
	Other	7 (5)		6 (12)		0 (0)		1 (2.2)	

^a^Values were rounded up using the tenths to the nearest whole number.

^b^Italicized values indicate the largest value.

### Bibliometrics

#### SciVal

Data from SciVal for all 143 scholars revealed a median of 17 publications or 3.4 publications per year on average at the 5-year time point. Impact metrics showed a midpoint of 26.16 citations per year or an average of 130.8 citations over 5 years. KL2 scholars were cited approximately 7 times more than other researchers in their field. In addition, 44.01% (1558/3540) of the publications were in the top 10th percentile of the journals. In terms of collaboration, KL2 scholars published work with researchers from other US institutions over 2 times more than others in their research fields. Contrary to the overall trends, investigating scholars at the institution level presented some differences (Table 3). The KL2 scholars of Institution C had a significantly higher citation rate per publication than the other institutions (H_2_=12.35; *P=*.002). Institution A had a significantly lower rate of nationally field-weighted collaboration than did the other institutions (H_2_=70.49; *P*<.001). No other 5-year significant differences were reported.

Data from SciVal for 112 scholars showed an increase in publication rate at the 8-year time point, with scholars from all institutions publishing an average of 3.75 publications per year, up from 3.4 at the 5-year time point. The mean citation rate also slightly increased from 26.16 at the 5-year time point to 26.44 at the 8--year time point. The percentage of publications in the top 10th percentile of journals decreased by less than 1% to 43.34% (2658/6132). Rates of collaboration increased, with KL2 scholars at 8 years publishing with researchers across the nation 3.5 times more than other researchers in their field. Results at the institutional level for the 8-year time point were similar to those at the 5-year time point (Table 3). Institution C scholars had a significantly higher rate of citations per publication (H_2_=10.12; *P=*.006), and scholars of Institution A had a significantly lower rate of field-weighted national collaboration (H_2_=65.08; *P*<.001; n=112; Institution A: n=37; Institution B: n=37; and Institution C: n=38).

Regarding demographics, outcomes from SciVal metrics reported that male scholars published significantly more work at the 5-year time point than female scholars (U=1114.50; *P*=.008; Table 4). There were no differences between the scholar race and any reported bibliometric outcomes. Scholars with an MD degree had significantly higher field-weighted citation indices than scholars without an MD degree (U=1202.50; *P*=.04).

**Table 3:** Postmatriculation SciVal bibliometric summary (medians reported).

Bibliometric	Value, median									
	All institutions		Institution A			Institution B			Institution C	
	5-year value (n=143)	8-year value (n=112)	5-year value (n=143)	8-year value (n=112)	5-year value (n=143)		8-year value (n=112)	5-year value (n=143)		8-year value (n=112)
FWCI^a^	7.16	12.4	6.79	10.2	6.8		12.6	*8.4^b^*		*14.2*
Scholarly output	17	30	15	27	15		26.5	*22*		*40.5*
Citations per publication	130.8	211.5	93.9	161.3	109.9		174.4	*174.1*		*288*
PPTPJ^c^ (%)	44	43.3	40.9	43.2	39		41.9	*48.5*		*45.8*
NFWC^d^	2.07	3.6	0.78	65	*3.4*		*5.7*	2.9		4.9

^a^FWCI: field-weighted citation impact.

^b^Italicized values indicate the largest values.

^c^PPTPJ: percentage of publications in the top 10th percentile of journals.

^d^NFWC: National Field-Weighted Collaboration.

**Table 4:** SciVal bibliometric outcomes by demographic groups (medians reported).

Bibliometric	Value, median																		
	Gender							Race							Degree				
	5-year value			8-year value			5-year value				8-year value			5-year value				8-year value	
	Male	Female	Male		Female	White			People of color	White		People of color	MD			Non-MD	MD		Non-MD
FWCI^a^	*8.1^b^*	6.3	*14*		10	7			*8*	11		*13*	*8*			6	*12*		9
Scholarly output	*23*	15	*35*		26	*18*			12	*30*		29	*20*			15	*35*		25
Citations per publication	*144*	115	*200*		185	125			*167*	185		*204*	*144*			109	192		*193*
PPTPJ^c^	*45*	39	*43*		41	42			*49*	40		45	*47*			39	*44*		36
NFWC^d^	*2.2*	1.8	3.6		3.6	*2.1*			1.6	*3.9*		2.2	*2.3*			1.3	*4*		2.5

^a^FWCI: field-weighted citation impact.

^b^Italicized values indicate the largest value.

^c^PPTPJ: percentage of publications in the top 10th percentile of journals.

^d^NFWC: National Field-Weighted Collaboration.

#### iCite

Data from iCite only provided a comprehensive report from all the years included in this study, as illustrated in Table 5. KL2 publications from all institutions were cited twice as much as other researchers in their fields, earning an average of 4.5 citations per year. The average NIH percentile was 53%, indicating that KL2 publications had an RCR higher than 53% of all NIH-funded publications [17]. At the institutional level, Institution A reported significantly lower results for the average field-weighted citation rate (H_2_=8.96; *P*=.01). No other significant results were reported.

**Table 5:** iCite bibliometric outcomes by institution (medians reported).

Bibliometric	Value, median			
	All institutions	Institution A	Institution B	Institution C
Average RCR^a^	1.67	1.51	1.72	*1.78^b^*
Average citations per year	3.47	2.76	*3.84*	3.82
Average field citation rate	4.05	3.79	*4.27*	4.09
Average NIH^c^ percentile	52.91	51.86	*54.17*	52.69

^a^RCR: relative citation ratio.

^b^Italicized values indicate the largest value.

^c^NIH: National Institutes of Health.

#### Federal Follow-on Funding

Grant analysis of KL2 scholars at all 3 institutions indicated that 44.1% (63/143) of KL2 scholars received federal funding within 5 years postmatriculation. At the 8-year time point, 51.7% (74/143) of scholars had achieved federal funding as a principal investigator, coprincipal investigator, or coinvestigator. Significant differences between institutions were found at the 5-year, 8-year, and overall funding rates using the chi-square test (Table 6). Scholars from Institution B were more likely to have received federal funding than scholars at Institution A or C at the 5-year (χ^2^_2_=15.28; *P*<.001) and 8-year (χ^2^_2_=7.07; *P=*.03) time points. Investigating federal grant funding by scholar characteristics showed no significant differences between gender, race, or degree at the 5-year or 8-year time points. Male scholars in our sample appeared to receive federal funding at higher rates than females, but this did not reach significance (χ*^2^*_2_=3.56; *P*=.06).

**Table 6:** Scholars with extramural federal funding by institution.

Time point	All institutions (N=143), n (%)	Institution A (n=50), n (%)	Institution B (n=48), n (%)	Institution C (n=45), n (%)
5-year	63 (44.1)	16 (32)	*31 (64.5^a^)*	13 (28.9)
8-year	74 (51.7)	24 (48)	*32 (66.7)*	18 (40)
Overall	74 (51.7)	24 (48)	*32 (66.7)*	19 (42.2)

^a^Italicized values indicate the largest value.

## Discussion

### Principal Findings

Overall, this study emphasizes the high level of scholarly productivity, impact, collaboration, and funding achieved by KL2 scholars. This study also provides insights into the utility of both bibliometric and federal follow-on funding to evaluate CTR training success, especially for measuring the scholarly work of training participants. Bibliometric data provide a better understanding of the impact of research publications produced by KL2 scholars. Federal funding data demonstrate the extent to which KL2 scholars are receiving subsequent federal funding and therefore successfully sustaining their research.

### Bibliometrics

On the basis of previous experience of applying bibliometrics to CTSA performance assessment [8-12], this study adopted a proprietary research analytics tool, SciVal, and a publicly available federal government–developed bibliometric tool, iCite, to investigate the productivity and citation impact of KL2 scholars across 3 institutions. Both SciVal and iCite metrics showed that the examined research publications of KL2 scholars had a more significant citation influence than those published simultaneously by other researchers in the same fields. For instance, SciVal metrics (eg, field-weighted citation impact [FWCI] and percentage of publications in the top 10th percentile of journals) showed that, on average, at 5 years, KL2 scholars were cited almost 7 times more than other researchers in their field. Approximately half of their articles were published in the top 10th percentile of the world's journals, which indicates the distinguishing influence of CTSA-supported KL2 scholars. Similarly, the RCRs generated by iCite disclosed that the KL2 scholars at 3 institutions received almost twice as many citations per year as other researchers in their fields, which is consistent with results reported in a previous study [12]. The difference of citation impact results produced by SciVal (FWCI) versus iCite (RCR) is associated with varied time ranges and citation tracking scopes that these two tools examined. In this study, FWCI results were generated at the 5- and 8-year time point, whereas the RCR of an institution was the average of all included publications from 2005 to 2019. In addition, the Scopus database, from which SciVal data are derived, is one of the largest citation databases in the world and covers the MEDLINE database, which is the primary component of PubMed in addition to thousands of international publishers. Consequently, the citation counts and FWCIs provided by SciVal or Scopus are often higher than those generated by iCite, which only tracks citation counts within the NIH Open Citation Collection, including MEDLINE, PubMed Central, and CrossRef [23].

Furthermore, KL2-scholars demonstrated significant collaborations with researchers across the United States, with coauthorship occurring 2 times more than others in their research fields at the 5-year time point and 3.6 times more at the 8-year time point, as measured by the National Field-Weighted Collaboration score generated by SciVal. Therefore, our results confirm the feasibility of applying bibliometrics to assess scholarly work supported by CTSA programs and corroborate the effectiveness of three CTSA programs in supporting KL2 scholars for translational research [8,9].

Nevertheless, a subanalysis highlighted a few critical demographic differences between scholars across institutions. Male scholars published significantly more than female scholars did at 5 years postmatriculation; however, this difference was not observed at the 8-year time point. Previous research confirms the difference in publications by males, but limited research has investigated any long-term differences [24]. Our results suggest that differences over time may be a critical point where female scholars reduce this gap, which may arise from a variety of personal commitments that impact the pace of the career of a female scientist. At 8 years postmatriculation, scholars with an MD degree had a significantly higher FWCI than those without an MD degree. These demographic differences should be further studied to better understand the role the scientific field plays in supporting scholarly work across different scientific areas.

Similarly, bibliometrics brought institutional differences to our attention (Multimedia Appendix 1). We found that KL2 scholars at Institution C had a significantly higher rate of citations per publication. In comparison, Institution A had a significantly lower rate of National Field-Weighted Collaboration than the other 2 institutions. These cross-institutional differences emphasize the need to invest in the standardization of measures (eg, bibliometrics), improving the ability to evaluate scholarly success across the nation. In previous research, CTSA hubs reported various techniques for reporting and tracking publications [8,9,25]. In addition, differences in publication characteristics have been shown to impact bibliometric outcomes (eg, reviews are usually cited more than original articles; open-accessible articles are cited more than nonopen accessible ones), such as in journal coverage of bibliographic databases, and the size and establishing time of CTSA programs [9,10,25]. Therefore, although it is feasible to use bibliometrics to analyze the scholarly output and influence of multiple CTSA programs, there are complications in applying citation metrics, interpreting results, and benchmarking the performance of multi-institutional programs. Future research should consider the role of the program-level characteristics outlined in the Multimedia Appendix 1.

### Federal Follow-on Funding

Our analysis reveals that federal funding award rates for KL2 scholars are higher than the national average of 20.2% [26]. Previous research reported a 34% R01 funding rate for KL2 scholars 6 years postmatriculation [27]. Our study included R01 equivalent awards, but nonetheless suggests that the federal funding rate for KL2 scholars in this sample (63/143, 44.1% at 5 years) may be slightly higher than other institutions. The data also show that male KL2 scholars are more likely to receive federal funding than their female counterparts; however, this was not statistically significant. Previous studies examining gender differences in obtaining grant funding have been mixed. There were mitigating effects by both the type of training and the length of time following training [21,22,28]. In our analysis, we investigated the attainment of any NIH funding, and thus, we might have picked up additional funding mechanisms not examined in previous research. To our knowledge, none of the studies, including ours, has examined funding from foundations or other sources. This exclusion is a limitation and potential area of further research, especially to better understand the underlying factors associated with male KL2 scholars having a higher likelihood of receiving follow-on funding.

### Limitations

The limitations of this study include programmatic differences between the 3 institutions, differences in scholarly scientific fields, and grant data. We intended to investigate the scholarly success of a multi-institutional sample of KL2 scholars and to identify appropriate and feasible methods that could be used across institutions. However, the analysis of these measures revealed that performance on certain metrics could be linked to institutional characteristics. Perhaps the most considerable difference between these three KL2 programs is the length of KL2 funded training time. Each institution had a different length of funding, ranging from 2 to 4 years, provided to scholars. Other programmatic distinctions, such as variations in grant writing support, also exist. Future research should identify which program characteristics are related to the outcomes studied here. Other possible analyses include investigating the impact of scholarly field and degree on outcomes given the differences between MDs and PhDs that we highlighted in our analyses.

### Conclusions

This study emphasized the use of bibliometrics and federal follow-on funding as necessary evaluation measures for assessing scholarly productivity, impact, collaboration, and funding achieved by KL2 scholars. We have shown that evaluators can use these metrics to evaluate CTR training programs that focus on scholarly productivity as a critical outcome. These methods can be used to complement existing evaluation strategies to demonstrate program performance. The findings of this study highlight the need to better understand barriers and facilitators of scholarly productivity. Institutions should consider similar subanalyses within their evaluations to explore equity within their programs. In addition, there is a need to investigate the impact of programmatic components and best practices that yield high follow-on funding rates. Program-level goals within and among institutions influence funding outcomes, scholarly productivity, and collaboration. Identifying these differences will enhance the specificity of KL2 program evaluations. CTR training programs, such as CTSA KL2 programs, can benefit from the findings of this and future analysis as they continuously adapt their program strategy to support the early-stage career development of CTR investigators.

## Appendix

Multimedia Appendix 1 KL2 program comparison across 3 Clinical and Translational Science Award hubs.

## Abbreviations

CTR: clinical and translational research
CTSA: Clinical and Translational Science Award
FWCI: field-weighted citation impact
NCATS: National Center for Advancing Translational Science
NIH: National Institutes of Health
RCR: relative citation ratio
RePORTER: Research Portfolio Online Reporting Tools Expenditures and Results
